# The Effect and Mechanism of Tamoxifen-Induced Hepatocyte Steatosis *in Vitro*

**DOI:** 10.3390/ijms15034019

**Published:** 2014-03-05

**Authors:** Fei Zhao, Ping Xie, Jiali Jiang, Lingqiang Zhang, Wei An, Yutao Zhan

**Affiliations:** 1 Department of Gastroenterology, Beijing Tongren Hospital, Capital Medical University, Beijing 100730, China; E-Mails: fayefly.future@163.com (F.Z.); jiangjl@trhos.com (J.J.); 2 State Key Laboratory of Proteomics, Beijing Proteome Research Center, Beijing Institute of Radiation Medicine, Beijing 102206, China; E-Mails: xpxp922@163.com (P.X.); zhanglq@bmi.ac.cn (L.Z.); 3 Department of Cell Biology, Municipal Laboratory for Liver Protection and Regulation of Regeneration, Capital Medical University, Beijing 100069, China; E-Mail: anwei@ccmu.edu.cn

**Keywords:** tamoxifen, HepG 2 cells, non-alcoholic fatty liver disease, triglyceride

## Abstract

The aim of this study was to determine the effect and mechanism of tamoxifen (TAM)-induced steatosis *in vitro*. HepG 2 (Human hepatocellular liver carcinoma cell line) cells were treated with different concentrations of TAM for 72 h. Steatosis of hepatocytes was determined after Oil Red O staining and measurement of triglyceride (TG) concentration. The expressions of genes in the TG homeostasis pathway, including sterol regulatory element-binding protein-1c (SREBP-1c), peroxisome proliferator-activated receptor γ (PPARγ), CCAAT/enhancer-binding protein α (C/EBPα), fatty acid synthase (FAS), acetyl-CoA carboxylase (ACC), stearoyl-CoA desaturase (SCD), carnitine palmitoyltransferase 1 (CPT1) and microsomal triglyceride transfer protein (MTP), were examined using quantitative real-time PCR and Western blot analysis. Cell proliferation was examined using the cell counting kit-8 (CCK-8) assay. We found that hepatocytes treated with TAM had: (1) induced hepatocyte steatosis and increased hepatocyte TG; (2) upregulation of SREBP-1c, FAS, ACC, SCD and MTP mRNA expressions (300%, 600%, 70%, 130% and 160%, respectively); (3) corresponding upregulation of protein expression; and (4) no difference in HepG 2 cell proliferation. Our results suggest that TAM can induce hepatocyte steatosis *in vitro* and that the enhancement of fatty acid synthesis through the upregulations of SREBP-1c and its downstream target genes (*FAS*, *ACC* and *SCD*) may be the key mechanism of TAM-induced hepatocyte steatosis.

## 1. Introduction

Breast cancer is the most common cancer in women around the world [1,2]. About 12.7% of American women develop breast cancer in their lifetime [3]. Tamoxifen (TAM) has been widely used for many decades as the “gold standard” adjuvant treatment for patients with breast cancers [4,5]. However, long-term use of TAM is associated with various complications, including hypoglycemia, hypertriglyceridemia, changes in plasma cholesterol levels and liver diseases, such as nonalcoholic fatty liver disease (NAFLD) [6]. It has been shown that 43% of breast cancer patients treated with TAM develop steatosis within the first two years of treatment, and overweight, women can also have steatohepatitis and cirrhosis [7]. Thus, prevention and treatment of NAFLD in breast cancer patients require the elucidation of the mechanism of TAM-induced NAFLD. However, previous studies on this subject have mainly used animal models, and the results are inconsistent. Although human primary hepatocyte culture is a suitable model of human liver [8,9], the scarcity of liver samples greatly hinders its application. In the present study, we used an alternative model of human liver, the human hepatocyte-derived cell line HepG2 [10], to explore the effect and cellular mechanism of TAM-induced hepatocyte steatosis *in vitro*.

## 2. Results

### 2.1. TAM Induced Lipid Accumulation in HepG2 Cells

**Figure 1.**
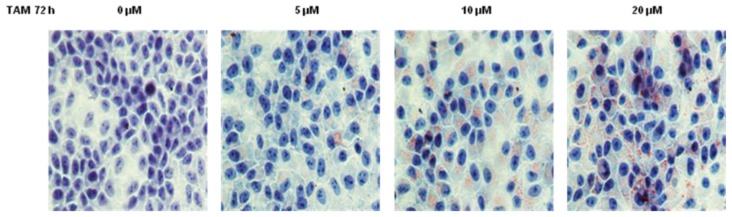
The effect of increasing concentrations of tamoxifen (TAM) on intracellular lipid accumulation in HepG2 (Human hepatocellular liver carcinoma cell line) cells stained with Oil Red O. Hepatocytes were incubated with 0, 5, 10 and 20 μM of TAM for 72 h, respectively, and then collected for Oil Red O staining. Representative images of Oil Red O stained cells treated with different concentrations of TAM are shown. Cells were examined under a light microscope at a magnification of 400×.

**Figure 2.**
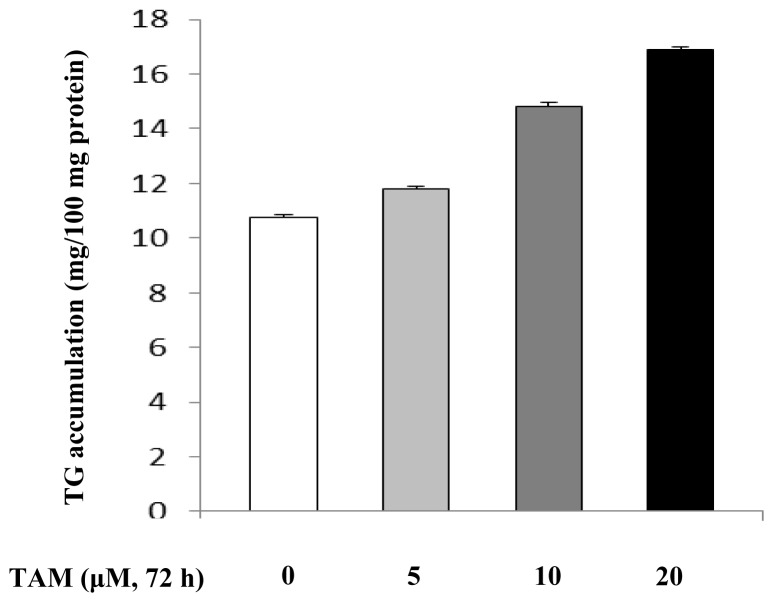
The effect of increasing concentrations of TAM on triglyceride (TG) accumulation in HepG2 cells. Hepatocytes were incubated with 0, 5, 10 and 20 μM of TAM for 72 h, respectively, and then collected for TG measurement. TG concentration was normalized with protein content. Experimental procedures are shown in the Materials and Methods section. Data are expressed as the means ± SD (*n* = 6).

In order to investigate whether TAM induced hepatocyte steatosis, we treated HepG2 cells with various concentrations of TAM for 72 h. Lipid accumulation was examined after Oil Red O staining. As shown in Figure 1, HepG2 cells treated with TAM accumulated significant amount of lipid droplets in a dose-dependent manner. To evaluate the effect of TAM on lipid accumulation quantitatively, we next measured triglyceride (TG) concentration in cell lysate. Consistently, 5 μM of TAM showed little effect on TG accumulation, while significant increases in TG were observed in cells treated with 10–20 μM of TAM (Figure 2). The average intracellular TG level in cells treated with 20 μM of TAM was 57% higher than that in the control cells.

### 2.2. Expression of Genes Regulating Lipid Metabolism

**Figure 3.**
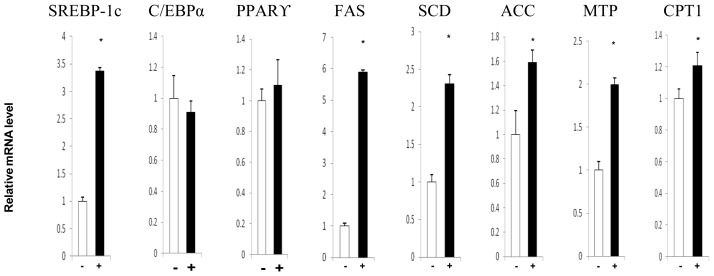
Effect of TAM on the mRNA expression of genes regulating lipid metabolism in HepG2. The effects on mRNA expressions of *SREBP*-1c, *C/EBP*α, *PPAR*γ, *FAS*, *SCD*, *ACC*, *MTP* and *CPT*1 are shown. Hepatocytes were incubated with 20 μM TAM for 72 h and then collected for RNA analysis. The experimental procedures are shown in the Materials and Methods section. Data are expressed as the means ± SD (*n* = 6); ^*^ *p* < 0.05.

According to the results obtained in the abovementioned experiment, we used 20 μM of TAM to investigate the effects of TAM on gene expression in hepatocytes (Figure 3). Quantitative assessment of the expression of genes involved in lipid metabolism showed that the increase of steatosis induced by TAM was associated with a 300% upregulation of the expression of sterol regulatory element-binding protein-1c (*SREBP*-1c), a determinant of fatty acid biosynthesis. *SREBP*-1c is a transcription factor that promotes the expression of lipogenic genes, such as fatty acid synthase (*FAS*), acetyl-CoA carboxylase (*ACC*) and stearoyl-CoA desaturase (*SCD*). We also observed that the expressions of *FAS*, *ACC* and *SCD* in cells treated with TAM increased by 600%, 70% and 130%, respectively. In contrast, the expressions of peroxisome proliferator-activated receptor-γ (*PPAR*-γ) and CCAAT/enhancer-binding protein α (*C/EBP*α), two other transcription factors controlling lipogenic gene expression, were unchanged. These findings indicated that TAM might enhance fatty acid synthesis through the *SREBP-*1c pathway. To determine whether or not TAM was associated with fatty acid β-oxidation, we investigated the expression of carnitine palmitoyltransferase 1 (*CPT*1), the rate-limiting enzyme of the mitochondrial fatty acid β-oxidation pathway, and the results showed that there was no change in *CPT*1 expression in response to TAM. Interestingly, the mRNA level of microsomal triglyceride transfer protein (*MTP*) (a TG-transportation related gene) was increased by 160% in HepG2 cells after TAM treatment. The upregulation of *MTP* might be the compensation response to TAM-induced increase in TG concentration.

### 2.3. Regulation of Proteins Involved in Lipid Homeostasis by TAM

**Figure 4.**
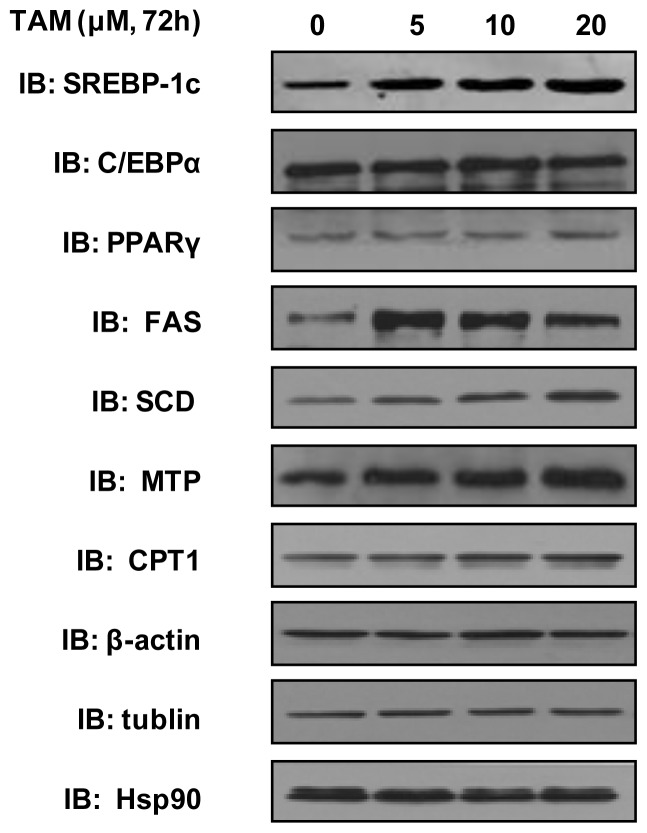
The effect of increasing concentrations of TAM on the expressions of proteins (SREBP-1c, C/EBPα, PPARγ, FAS, SCD, MTP and CPT1) involved in lipid homeostasis in HepG2. Hepatocytes were incubated with 0, 5, 10 and 20 μM of TAM for 72 h, respectively, and then collected for Western blot analysis. Experimental procedures are shown in the Materials and Methods section (IB: immunoblot).

We next examined the expressions of proteins induced by TAM using Western blot analysis (Figure 4). The proteins encoded by genes associated with fatty acid synthesis, including SREBP-1c, FAS, ACC and SCD, were upregulated in dose-dependent fashions. The protein levels of PPARγ and C/EBPα remained unchanged. Similarly, TAM did not alter the expression of CPT1 protein, but induced the expression of MTP protein. Together, these results suggested that the TAM-induced accumulation of TG in HepG2 cells was due to the increase in fatty acid synthesis.

### 2.4. Effects of TAM on HepG2 Proliferation

**Figure 5.**
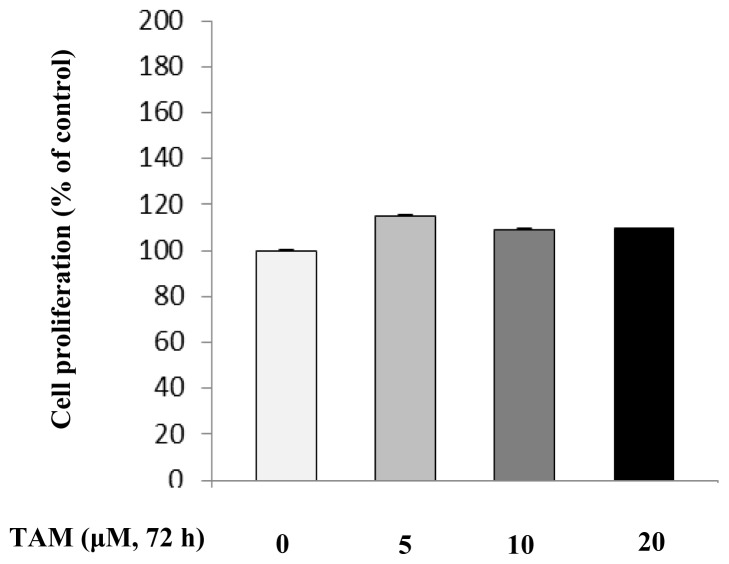
The effect of increasing concentrations of TAM on the cell proliferation of HepG2. Hepatocytes were incubated with 0, 5, 10 and 20 μM of TAM for 72 h, respectively, and then collected for cell viability assay. Experimental procedures are shown in the Materials and Methods section. Data are expressed as the means ± SD (*n* = 6). No statistically significant difference in cell proliferation was observed when cells were treated with 5–20 μM of TAM.

To further investigate the effect of TAM on TG, the proliferation activity of TAM should be evaluated. We assessed the effect of TAM on HepG2 cell proliferation by incubating the cells with various doses of TAM for 72 h and then measuring cell viability using the cell counting kit-8 (CCK-8) assay. Hepatocytes incubated with 5–20 μM of TAM showed no marked changes in proliferation (Figure 5). Therefore, the increase in TG concentration in cells treated with 5–20 μM of TAM was not due to increased cell proliferation. However, we observed that a high dose (over 20 μM) of TAM had a cytotoxic effect (data not shown) on HepG2 cells.

## 3. Discussion

Several clinic studies have indicated that TAM treatment is associated with the development of NAFLD [11–14]. Animal experiments have also confirmed that TAM can induce NAFLD [15]. In the present study, we explored the effect of TAM on hepatocyte steatosis and demonstrated that TAM induced hepatocyte steatosis *in vitro*. The results of our study provided direct evidence of TAM-induced hepatocyte steatosis.

NAFLD is characterized by the accumulation of hepatocyte TGs, which are formed from the esterification of free fatty acids (FFAs) and glycerol. FFAs arise in the liver from three distinct sources: (a) recirculation of non-esterified fatty acids from peripheral tissues (some from adipose tissues and some from skeletal muscle); (b) *de novo* lipogenesis (DNL) within hepatocytes; and (c) dietary sources. Adipose tissue is the main source of liver FFAs. Approximately 60% of liver TGs are derived from FFA influx from the adipose tissue; 25% are from DNL, and 15% are from diet [16]. In contrast, FFA may be utilized either through β-oxidation via re-esterification and stored as TGs in lipid droplets, or packaged and exported as very low density lipoprotein. Hence, hepatic fat accumulation can occur as a result of increased fat synthesis, decreased fat oxidation and/or decreased fat export.

SREBPs belong to the basic helix-loop-helix-leucine zipper family of transcription factors [17]. SREBPs have three members: SREBP-1a, SREBP-1c and SREBP-2. SREBP-1c is the major (90%) isoform that controls fatty acid synthesis in the liver [18]. SREBP-1c transcriptionally activates all genes required for lipogenesis [19,20], such as ATP-citrate lyase (ACL), ACC, FAS, SCD and glyceraldehyde-3-phosphate acyltransferase (GPAT) [21,22]. Previous studies have shown contradictory results on TAM-induced expressions of SREBP-1c and its downstream genes in animal fatty liver. Lelliott *et al*. have found that the level of SREBP1 mRNA in TAM-treated rat liver does not change in response to TAM treatment, but the levels of FAS, SCD1 and ACC mRNAs in TAM-treated rat livers are lower than those in the control group [15]. Cole *et al*. have reported that there are no differences in the levels of mice liver SREBP1 and ACC mRNAs between control mice and TAM-treated mice, but the level of FAS mRNA in TAM-treated mice is increased by 200%; the level of SCD1 mRNA in TAM-treated mice is reduced by 60% [7]. Gudbrandsen *et al*. have found that TAM does not affect the activities and mRNA levels of rat liver SCD1, but TAM can decrease the activities of rat liver ACC and FAS [23]. In the present study, we found that the mRNA and protein expressions of SREBP-1c, FAS, SCD1 and ACC increased in response to TAM treatment in HepG2 cells. Our data suggested that TAM induced the accumulation of hepatocyte TGs by upregulating SREBP-1c transcriptional activity and its downstream lipogenesis genes.

PPAR-γ and C/EBP-α are two other major transcription factors regulating adipogenesis [23]. FAS and SCD1 are downstream target genes of PPAR-γ. SCD1 is a downstream target gene of C/EBP-α [24]. We found that TAM failed to affect the mRNA and protein expressions of PPAR-γ and C/EBP-α. These results suggested that steatosis was not induced by TAM treatment through the regulation of the two transcription factors, PPAR-γ and C/EBP-α.

The observation that increased hepatic triacylglycerol level after TAM treatment is accompanied by decreased serum triacylglycerol level [24] suggests that the assembly or secretion of very low density lipoprotein (VLDL) from the liver may be inhibited by TAM. VLDL is a large particle containing apolipoprotein B (apoB) and various lipids, including TG, free cholesterol and cholesteryl ester. It is assembled in endoplasmic reticulum in hepatic cells [25]. ApoB is the essential protein component of VLDL [26]. Animal studies have shown that TAM has no effect on hepatic apoB level [23]. Thus, TAM does not affect liver VLDL content. MTP is an essential factor for VLDL assembly and secretion. It binds to various lipid molecules, including TG, and then presents those lipids to apoB [27]. In this study, we observed that TAM did not inhibit MTP mRNA and protein expression in HepG2 cells. Thus, we consider that increased TG accumulation in HepG2 cells treated with TAM may not be associated with the intrahepatic to extrahepatic VLDL assembly, secretion and TG transport. In contrast, we found that TAM increased MTP mRNA and protein expression in HepG2 cells. It is possible that TAM induced TG accumulation, which stimulated MTP mRNA and protein expression through a feedback regulation mechanism.

Is the increased triacylglycerol content in hepatocyte with TAM associated with the reduction of fatty acid β-oxidation in mitochondrial? CPT1 is the rate-limiting enzyme of the mitochondrial fatty acid β-oxidation pathway [28,29]. Cole *et al*. have found that there are no differences in the levels of CPT1 mRNA in liver between control mice and TAM-treated mice [6]. Gudbrandsen *et al*. have reported that TAM does not affect the activities and mRNA levels of rat liver CPT1 nor change the levels of acetyl-CoA and propionyl-CoA originated from β-oxidation in liver [23]. In the present *in vitro* study, TAM failed to affect CPT1 mRNA and protein expression in hepatocytes. Collectively, these data indicated that TAM accelerated the accumulation of TG in hepatocytes, and this might not be associated with the decrease in mitochondrial fatty acid β-oxidation.

In this study, we did not observe the effect of TAM on hepatocyte proliferation. This suggested that the increase of TG caused by TAM was not due to hepatocyte proliferation. Because TAM is an estrogen antagonist, the TAM-induced hepatocyte steatosis might be due to the inhibition of estrogen signaling. Further studies are needed to elucidate this possibility.

TAM can be converted into five metabolites in HepG2 cells: *N*-desmethyl-TAM, 4-hydroxy-TAM, TAM-*N*-oxide, 3,4-dihydroxy-TAM and 4-hydroxy-TAM-*N*-oxide. *N*-desmethyl-TAM is a major metabolite of TAM and is mainly catalyzed by CYP3A4, the most highly expressed isozyme in P450 subfamilies in HepG2 cells. Which metabolite plays a key role in TAM-induced hepatocyte steatosis is still unknown. As a major metabolite, *N*-desmethyl-TAM may play a major role in the pathogenesis of hepatocyte steatosis caused by TAM [30].

## 4. Materials and Methods

### 4.1. Reagents and Antibodies

TAM and Oil Red O were purchased from Sigma (St. Louis, MO, USA). The cell counting kit-8 was obtained from Dojindo Molecular Technologies (Kumamoto, Japan). The TG assay kit was obtained from Bioassay Systems (Hayward, CA, USA). The fatty acid synthase antibody was purchased from Santa Cruz. The anti-SREBP1 antibody, anti-MTP antibody, anti-CPT1A antibody, anti-ACC antibody and anti-SCD antibody were from Abcam. The PPARγ antibody was obtained from Cell Signaling Technology (Boston, MA, USA). Antibodies to C/EBPα and GAPDH were from Epitomics and Marine Biological Laboratory (Beijing, China), respectively. All other reagents were of analytical grade.

### 4.2. Cell Culture and Treatments

HepG2 cells were preserved in the Institute of Infectious Disease, Ditan Hospital, Capital Medical University, and cultured in Dulbecco’s Modified Eagle’s medium (DMEM) (Hyclone, Boston, MA, USA) supplemented with 10% fetal bovine serum, penicillin (50 U/mL) and streptomycin (50 μg/mL) (Hyclone, Boston, MA, USA) at 37 °C in an atmosphere of 5% CO_2_. HepG2 cells were seeded in 6-well plates. At 24 h after cell seeding, TAM was added to cell cultures at 0, 5, 10 and 20 μM. The cells were further incubated for 72 h for RNA and protein analyses, as well as for the measurement of cellular lipid content.

### 4.3. Oil Red O Staining

HepG2 cells were grown on a coverslip, washed with phosphate-buffered saline and then fixed with 10% formalin solution for 5 min at room temperature. After fixation, cells were washed gently with 60% isopropanol and stained with the working solution of 0.5 g Oil Red O in 60% isopropanol for 15 min. The stained hepatocytes were washed with distilled water several times to remove unincorporated dye. Then, the samples were counterstained with hematoxylin for 5 min. Results were examined using a light microscope.

### 4.4. Intracellular Lipid Content Assessment Using TG Assay

HepG2 cells were pre-incubated in 25-cm^2^ cell culture flasks for 24 h and then cultured in DMEM with 0–20 μM of TAM, as described above. After 72 h of incubation, cells were scraped and transferred into an Eppendorf tube (1.5 mL) and centrifuged at 3000 rpm for 5 min. Cell pellets were washed with Phosphate-Buffered Saline (PBS) once, resuspended in 400 μL PBS buffer and transferred to a micro smashing tube for ultrasonication. After ultrasonication, the concentration of cellular TG was determined using an EnzyChrom™ triglyceride assay kit (Kampenhout, Belgium) and normalized with protein concentration, according to the protocol provided by the manufacturer.

### 4.5. RT-PCR and Real-Time PCR

HepG2 cells were harvested and total RNA was extracted using the TRIZOL reagent (Invitrogen, Grand Island, NY, USA), according to the manufacturer’s instructions. Reverse transcription of 3 μg total RNA was performed by combining 1 μL ReverTraAce-α-™ (Toyobo, Shanghai, China), 4 μL 5× buffer, 1 μL 10 pmol/μL oligo(dT) primer, 2 μL 10 mM dNTP mix, 1 μL 10 U/μL RNase inhibitor and water to 20 μL. Reaction mixtures were incubated at 30 °C for 10 min, 42 °C for 20 min, 99 °C for 5 min, 4 °C for 5 min and then diluted with 30 μL of water. Real-time PCR was performed using a Bio-Rad iQ5 PCR machine (Bio-Rad, Hercules, CA, USA). Each PCR mixture contained 0.5 μL of cDNA template and primers at a concentration of 100 nM each in a final volume of 25 μL of SYBR green reaction mix (Toyobo, Shanghai, China). Each PCR generated only the expected amplicon, as shown by the melting temperature profiles of the final products and by gel electrophoresis. Standard curves were calculated using cDNA to determine the linear range and PCR efficiency of each primer pair. Reactions were performed in triplicate, and the relative amount of cDNA was normalized to β-actin. Primers used in these analyses are shown in Table 1.

### 4.6. Western Blot Analysis

Cell lysates were prepared in HEPES (*N*-(2-hydroxyethyl) piperazine-*N*′-2-ethanesulfonic acid) lysis buffer (20 mM HEPES, 50 mM NaCl, 0.5% Triton X-100, 1 mM NaF and 1 mM dithiothreitol). Lysates were examined using the indicated primary antibodies followed by detection with the related secondary antibody with a Super Signal chemiluminescence kit (Thermo Fisher, Boston, MA, USA).

### 4.7. Cell Viability Assay

Cell viability was measured using a tetrazolium salt (WST-8, C20H13N6NaO11S2)-based colorimetric assay in the cell counting kit-8 (CCK-8, Dojindo Molecular Technologies, Kumamoto, Japan). Briefly, cells were seeded in 96-well plates at an initial density of 1 × 10^5^ cells per well. After 24 h, the cells were washed with PBS, and the medium was replaced with the experimental medium with 0–20 μM of TAM. After 72 h of incubation, the spent medium was replaced with fresh medium containing 10 μL CCK-8 solution, and the plate was incubated for 1 h. Cell viability was measured by scanning with a microplate reader at 450 nm. Data were expressed as A450 (treated or control)-A450 (blank).

### 4.8. Statistical Analysis

All experiments were performed in centuplicate and repeated at least three times. Data are shown as the mean ± SD. The significance of differences was determined by one-way ANOVA using the SPSS 17.0 software (SPSS, Chicago, IL, USA). A value of *p* < 0.05 was considered statistically significant.

## 5. Conclusions

In the present study, we found that TAM induced hepatocyte steatosis *in vitro*. Our data indicated that the SREBP-1c transcription pathway played an important role in the regulation of TAM-mediated TG accumulation in hepatocytes. These data suggested that the inhibition of SREBP-1c might have therapeutic potential for TAM-reduced hepatocyte steatosis.

## Figures and Tables

**Table 1.** Primer sequences used for real-time RT (Reverse transcription)-PCR.

Genes	Primer sequences (5′-3′)	GenBank accession number
*SREBP*-1c	Forward CGGAACCATCTTGGCAACAGT	NM_001005291
	Reverse CGCTTCTCAATGGCGTTGT	
*C/EBPα*	Forward CGCCTTCAACGACGAGTTCCTG	NM_004364
	Reverse CGCCTTGGCCTTCTCCTGCT	
*PPAR* γ	Forward ACCAAAGTGCAATCAAAGTGGA	NM_138711
	Reverse ATGAGGGAGTTGGAAGGCTCT	
*FAS*	Forward AAGGACCTGTCTAGGTTTGATGC	NM_004104
	Reverse TGGCTTCATAGGTGACTTCCA	
*SCD*	Forward TTCCTACCTGCAAGTTCTACACC	NM_005063
	Reverse CCGAGCTTTGTAAGAGCGGT	
*ACC*	Forward ATGTCTGGCTTGCACCTAGTA	NM_198838
	Reverse CCCCAAAGCGAGTAACAAATTCT	
*MTP*	Forward ACAAGCTCACGTACTCCACTG	NM_000253
	Reverse TCCTCCATAGTAAGGCCACATC	
*CPT1*	Forward ATCAATCGGACTCTGGAAACGG	NM_001876
	Reverse TCAGGGAGTAGCGCATGGT	
*β-actin*	Forward CTGGGACGACATGGAGAAAA	NM_001101
	Reverse AAGGAAGGCTGGAAGAGTGC	
